# Chemotherapy‐driven expression of WNT ligands in bone marrow stromal cells contributes to chemoresistance in acute lymphoblastic leukaemia

**DOI:** 10.1111/bjh.70431

**Published:** 2026-03-17

**Authors:** Foteini Kalampalika, Amanda Jiménez‐Pompa, Raúl Sánchez‐Lanzas, Bela Patel, Miguel Ganuza

**Affiliations:** ^1^ Centre for Haemato‐Oncology, Barts Cancer Institute Queen Mary University of London London UK

**Keywords:** acute lymphoblastic leukaemia, bone marrow niche, chemoresistance, WNT pathway

## Abstract

Despite recent advances, treatment outcomes for adults with acute lymphoblastic leukaemia (ALL) remain poor. Although patients often exhibit an initial favourable response to chemotherapy, with substantial clearance of tumour cells, most patients eventually relapse. This indicates the persistence of a chemoresistant ALL subpopulation capable of driving disease regeneration. Growing evidence implicates interactions between leukaemia cells and the bone marrow (BM) niche in this process. Our findings show that BM‐derived mesenchymal stem cells (MSCs) and adipocytes (BMAds) promote chemotherapy resistance in ALL cells via activation of the wingless‐related integration site (WNT) signalling pathway. Chemotherapy‐treated co‐cultures of MSCs/BMAds and ALL cells exhibited upregulation of several WNT ligands in the stromal compartment. Notably, pharmacological inhibition of WNT signalling abrogated the stromal‐mediated chemoprotection and enhanced ALL cell apoptosis in vitro. In vivo, WNT inhibition in a *p185* ^BCR‐ABL^ *Arf* ^−/−^ B‐ALL mouse model sensitised leukaemia cells to chemotherapy, delaying relapse and extending survival. Collectively, these results support the therapeutic potential of WNT inhibitors as a strategy to block the cross‐talk between the BM stroma and leukaemic cells and reduce ALL chemoresistance.

## Graphical abstract

Chemotherapy‐induced WNT ligand secretion by bone marrow (BM) stroma drives acute lymphoblastic leukaemia (ALL) chemoresistance. WNT pathway inhibition disrupts this cross‐talk and restores therapeutic response, highlighting WNT inhibitors as a promising strategy to prevent relapse in ALL.

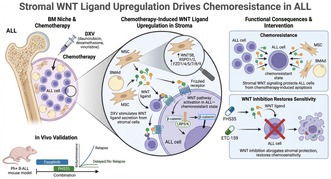

## INTRODUCTION

Over the past five decades, treatment of paediatric acute lymphoblastic leukaemia (ALL) has significantly improved, with ~80% of children achieving complete remission.^1^ However, prognosis remains poor for the ~20% who relapse and adult outcomes remain dismal despite recent advances in diagnosis and therapy.^2^ ALL can be genetically subtyped, with the breakpoint cluster region ‐ abelson murine leukemia 1 (*BCR‐ABL)* translocation (Philadelphia chromosome) representing a common oncogenic driver.^3^ Combining tyrosine kinase inhibitors (TKIs) such as imatinib or dasatinib with standard chemotherapies (dexamethasone, vincristine, daunorubicin and asparaginase) induces strong initial remissions.^4^ Yet, relapse ensues in most cases indicating persistence of a chemoresistant subpopulation.^5^

These chemo‐residual cells survive through genetic mutations, including BCR‐ABL kinase domain variants (e.g. T315I),^6^ and non‐genetic adaptations,^5^, ^7^, ^8^ often mediated by protective bone marrow (BM) microenvironment interactions.^2^, ^9^ The stromal signals underlying this resistance remain incompletely defined.^6^ Mesenchymal stem cells (MSCs), a key BM niche component, support multiple haematological malignancies^7^, ^10^, ^11^ and undergo treatment‐induced molecular changes that promote leukaemia survival and quiescence, including altered cytokine secretion, extracellular matrix remodelling, drug metabolism and increased adhesion molecule expression.^12^, ^13^ Disrupting MSC–leukaemia interactions holds therapeutic promise; for instance, targeting the Stromal Cell‐derived Factor (SDF) / CXC Chemokine Receptor 4 (CXCR4) pathway sensitises acute myeloid leukaemia (AML) to chemotherapy,^14^ but comparable strategies in ALL remain limited.^15^, ^16^, ^17^

Recent studies, including ours, highlight substantial BMAd accumulation following chemotherapy.^16^, ^18^ We showed that treatment‐induced adipocytes dominate the post‐therapy niche and promote ALL quiescence and reduced translation, enabling survival under chemotherapy.^15^ Adhesive interactions between ALL cells and adipocytes form a critical resistance axis, representing a potential therapeutic target.^15^

Although stromal‐induced changes in ALL cells have been examined,^11^, ^19^, ^20^, ^21^ we examined here chemotherapy‐induced stromal reprogramming as an independent driver of chemoresistance focusing on MSCs and MSC‐derived adipocytes (BMAds) co‐cultured with ALL cells. Chemotherapy upregulated WNT–β‐catenin pathway components (*RSPO1, RSPO2, WNT5A/B*, *PORCN*) in stromal cells. Pharmacological WNT inhibition using FH535 [a T‐cell factor (TCF)‐dependent transcription blocker] or ETC‐159 (a WNT ligand secretion inhibitor) sensitised B‐ALL cells to chemotherapy in vitro, while FH‐535 synergised with dasatinib in a *p185* ^BCR‐ABL^ *Arf* ^−/−^ B‐ALL mouse model,^22^ extending relapse‐free survival.

## MATERIALS AND METHODS

### Mice

All experiments involving mice were performed under Queen Mary University of London Veterinary oversight with UK Home Office authorisation and comply with all relevant ethical regulations regarding animal research. C57BL/6NCrl (Charles River #027), mice were employed (Charles River Laboratory, Harlow, UK) and housed in a pathogen‐free facility. Mice of both sexes were used.

### B‐ALL induction and in vivo treatments

C57BL/6 mice were transplanted with 2 × 10^5^ *Arf* ^−/−^ *BCR‐ABL p185* ^+^ *Luciferase* ^+^ pre‐B‐leukaemia initiating cells (LICs) by intravenous injection (day 0), as previously described.^23^ Cells were kindly donated by the laboratories of Martine Roussel and Charles Sherr (St. Jude Children's Research Hospital). Mice received dasatinib (10 mg/kg, oral gavage) once daily for 10 days, starting on day 7 post‐ALL infusion. A separate cohort received co‐treatment with WNT inhibitor FH535 (2.5 mg/kg, intraperitoneal) twice daily from days 7 to 21.

### Human bone marrow MSCs

Adult BM‐mononuclear cells (MNCs) were purchased from Lonza (2 M‐125C) (median age: 27 years; range: 21–47).

### Cell culture

Nalm6 cells were maintained in RPMI‐1640 + 10%FCS. *Arf* ^−/−^ *BCR‐ABL p185* ^+^ *Luciferase* ^+^ pre‐B‐LICs were cultured in RPMI‐1640 with 10% FCS and 55 μM β‐mercaptoethanol. Primary human MSCs were expanded in MEMα + 10%MSC‐qualified FBS.

### MSC adipogenic differentiation

Adipocyte differentiation was initiated at confluence using Lonza Adipogenesis Kit (#PT‐3004) following standard induction/maintenance cycles before switching to maintenance medium. Adipogenesis was confirmed by fatty acid‐binding protein 4 (FABP4) immunofluorescence and Oil Red O (ORO) lipid staining.

### BM stroma–ALL co‐cultures

After 19 days of adipogenesis, differentiated MSCs were co‐cultured with Nalm6 cells at a 1:2 ratio (stroma: ALL) in RPMI‐1640 + 10% FCS. Treatments included ETC‐159 (100 nM), FH535 (15 μM), LY294002 (50 μM) and DXV (8.3 ng/mL; 6.01 ng/mL daunorubicin, 2.01 ng/mL dexamethasone, 0.28 ng/mL vincristine).

### Flow cytometry

Data were collected on a BD LSR Fortessa I (FACSDiva v8.0.1) and analysed using FlowJo v10.8.0.

### Histopathology

Tissues were fixed in 10% buffered formalin (Sigma), paraffin‐embedded and stained with. haematoxylin and eosin staining (#760‐2021, #760‐2037; Roche) using a Leica Autostainer XL. Slides were mounted on a Leica CV5030.

### Cell population separation and RNA extraction from MSC/BMAd stroma

After 72‐h co‐culture under DXV, Nalm6 cells were removed by brief trypsinisation (1 min) using diluted trypsin/EDTA (50% diluted, Gibco), which enables efficient removal of Nalm6 cells from these co‐cultures.^15^ Stromal cells were subsequently collected with undiluted trypsin/EDTA.^15^ Separation efficiency was confirmed by flow cytometry. RNA was isolated using RNeasy Micro Kit (Qiagen).

### Quantitative real‐time PCR analysis

Total RNA (1 μg) was reverse transcribed (High‐Capacity cDNA Reverse Transcription Kit, Applied Biosystems) with random primers. Quantitative PCR was performed (C1000‐Touch thermal cycler, Bio‐Rad) using SSO‐Advanced Universal SYBR Green Supermix (Bio‐Rad).^24^ Primer sequences listed in Table S1. GAPDH served as normalisation control. Relative expression was calculated using the ΔΔCt method, with untreated samples set to 100.

### RNA sequencing and analysis

mRNA was isolated with poly‐T beads, fragmented, reverse‐transcribed and sequenced on an Illumina NovaSeq6000 (150 bp paired‐end). Reads were quality‐checked, trimmed and aligned to GRCh38 using HISAT2.^25^ Read counts were generated with ‘featureCounts’ against Ensembl GRCh38 (release‐114).^26^ DESeq2 (RStudio.2025.05.1; R4.4.3) analysed differential expression, adjusting for donor variation.^27^ Genes with <10 reads were excluded. Wald tests with Benjamini–Hochberg correction determined significance (*p*adj < 0.05). Log2FC was shrunken using apelgm.^28^ PCA and heat maps were produced using variance‐stabilised counts.

### Cell viability evaluation

Cell number and viability were determined with 0.4% Trypan Blue and a haemocytometer.

### Annexin V staining

Nalm6 cells were stained in Annexin V Binding Buffer (StemCell Technologies) with Annexin V‐APC (BioLegend) and 0.1 μg/mL DAPI and then analysed on a BD‐LSR‐Fortessa‐I.

### Bioluminescent imaging

Leukaemia progression was assessed using IVIS‐Lumina‐III (Revvity). Mice received intraperitoneally 100 mg/kg VivoGlo Luciferin 10 min before imaging under isoflurane. Imaging occurred on days 3, 5, 9, 12, 14, 18 and 21 post‐B‐ALL infusion (1‐min exposure, small binning). Total photon flux was quantified using Living Image v4.5.2 and normalised to 5 × 10^4^–2 × 10^6^ ph/s/cm^2^/sr. Relapse was defined as flux exceeding day‐3 baseline (1.3 × 10^6^ ph/s). Relapse‐free survival was analysed by Kaplan–Meier, and disease burden by photon flux, area under the curve and relapse kinetics.

### Statistical analysis

Data presented as means ± SD (*n* ≥ 3) and analysed using GraphPad‐Prism v10.3.1. Normality was assessed using Shapiro–Wilk. Statistical tests included unpaired/paired *t*‐tests, one‐way ANOVA with Dunnett's correction or Kruskal–Wallis test with Dunn's multiple comparisons; Kaplan–Meier curves were compared using the log‐rank test (Mantel–Cox). Significance was set at *p* < 0.05. No data were excluded, and no blinding, randomisation or prospective sample‐size calculations were used. Sample sizes are provided in figure legends.

### Critical reagents

Listed in Table S1.

## RESULTS

### MSC/BMAd stromal cells confer chemoprotection to Nalm6 ALL cells in vitro

**FIGURE 1:**
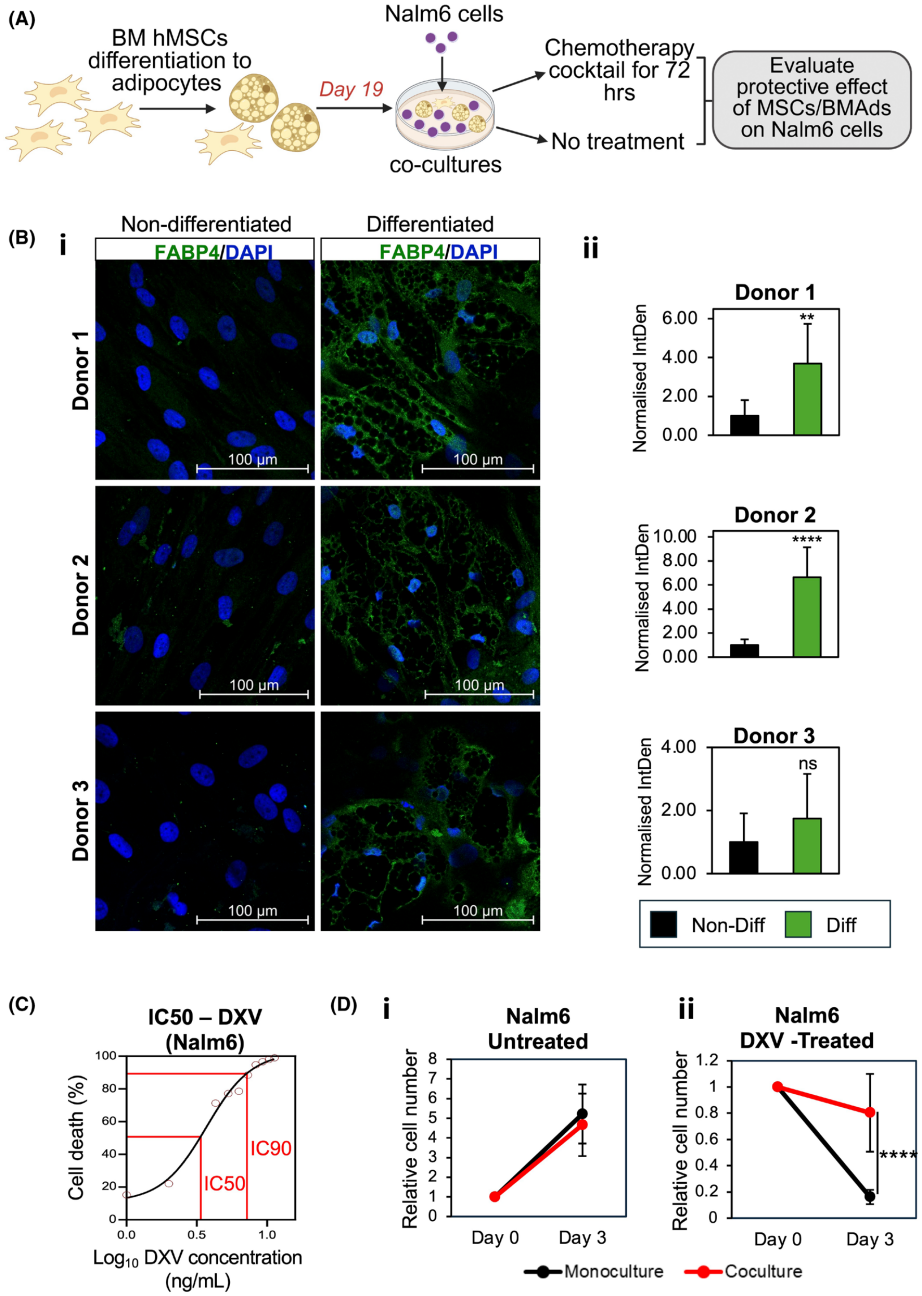
Bone marrow‐derived mesenchymal stem cells (MSCs) and adipocytes (BMAds) stromal cells mediate chemoprotection of Nalm6 cells in vitro. (A) Experimental schematic. Primary human MSCs derived from healthy donors were differentiated into adipocytes (BMAds) over 19 days. On day 19, Nalm6 acute lymphoblastic leukaemia (ALL)cells were added and co‐cultured with MSC/BMAds for 72 h in the presence or absence of daunorubicin, dexamethasone and vincristine (DXV) (8.3 ng/mL) (IC_90_). Nalm6 monocultures served as controls. Nalm6 cell numbers were quantified post‐treatment. (B) Quantification of FABP4 expression as a marker of adipogenic differentiation in MSCs. (Bi) Representative immunofluorescence images from three donors at day 19 of differentiation. Non‐differentiated MSCs served as controls. FABP4 (green) marks lipid‐filled adipocytes; nuclei are stained with DAPI (blue). Scale bar: 100 μm. (Bii) FABP4 signal intensity (integrated density, IntDen) normalised to DAPI‐positive nuclei. *N* = 8 technical replicates per donor from two independent preparations. (C) Dose–response curve of Nalm6 cells exposed to increasing concentrations of DXV for 72 h. Cell death was calculated relative to untreated controls. IC_50_ and IC_90_ were determined using a four‐parameter logistic regression (*R* ^2^ = 0.9892). (D) Nalm6 cell numbers in monoculture or co‐culture with MSC/BMAds under untreated conditions (Di) or after DXV treatment (8.3 ng/mL; IC_90_) (Dii). The effect of DXV on Nalm6 cell numbers was evaluated in three independent experiments, each encompassing four co‐cultures (each with MSC/BMAds derived from a different donor); six technical replicates were counted for monocultures and two to three technical replicates for each co‐culture. Data represent means ± SD. Statistical significance: ****p < 0.0001, **p < 0.01.

To investigate niche‐specific molecular drivers of ALL chemoresistance, we established co‐culture models of MSCs/BMAds with ALL cells under a clinically relevant chemotherapy regimen using common remission induction drugs (daunorubicin, dexamethasone and vincristine: DXV).^29^ Nalm6 cells, a human pre‐B‐ALL cell line harbouring the *DUX4/IGH* oncogenic fusion (present in ~7% of B‐ALL cases),^30^, ^31^ were co‐cultured with primary human MSCs differentiated into BMAds before ALL cell addition (Figure 1A).

MSCs from four healthy donors were differentiated into mature adipocytes^15^ with adipogenesis verified by Oil Red O (ORO) lipid staining and FABP4 immunofluorescence (Figure 1B, Figure S1). Lipid droplet accumulation and FABP4 expression varied among donors, reflecting donor‐dependent differences in adipogenic potential. In Nalm6 monocultures, the 72‐h half‐maximal inhibitory concentration (IC_50_) of DXV was 3.7 ng/mL (2.68 ng/mL daunorubicin, 0.9 ng/mL dexamethasone, 0.12 ng/mL vincristine), while IC_90_ was 8.3 ng/mL (6.01 ng/mL daunorubicin, 2.01 ng/mL dexamethasone, 0.28 ng/mL vincristine) (Figure 1C). The IC_90_ dose was selected to assess stromal responses and chemoprotective effects under strong cytotoxic pressure.

To evaluate stromal‐mediated chemoprotection, day‐19 MSC/BMAd cultures were co‐cultured with Nalm6 cells and treated with 8.3 ng/mL DXV (Figure 1A,D). Untreated and DXV‐treated Nalm6 monocultures served as controls. After 72 h, Nalm6 cells were harvested for counting by mild‐trypsinisation, as previously published.^15^ Purity of Nalm6 isolated cells was verified by flow cytometry (Figure S2). Cell numbers were similar between untreated monocultures and co‐cultures (Figure 1Di). In contrast, DXV‐treated co‐cultures displayed significantly higher Nalm6 counts than treated monocultures (*p* = 0.0139, *n* = 3) (Figure 1Dii). Consistent with prior studies,^15^, ^20^ these findings support that MSC/BMAd stroma confers chemoprotection to ALL cells. This stromal resistance model was therefore used to investigate stromal‐specific molecular mechanisms underlying ALL chemoresistance.

### DXV chemotherapy induces expression of WNT pathway activators in MSC/BMAd stromal cells co‐cultured with Nalm6 ALL cells

**FIGURE 2:**
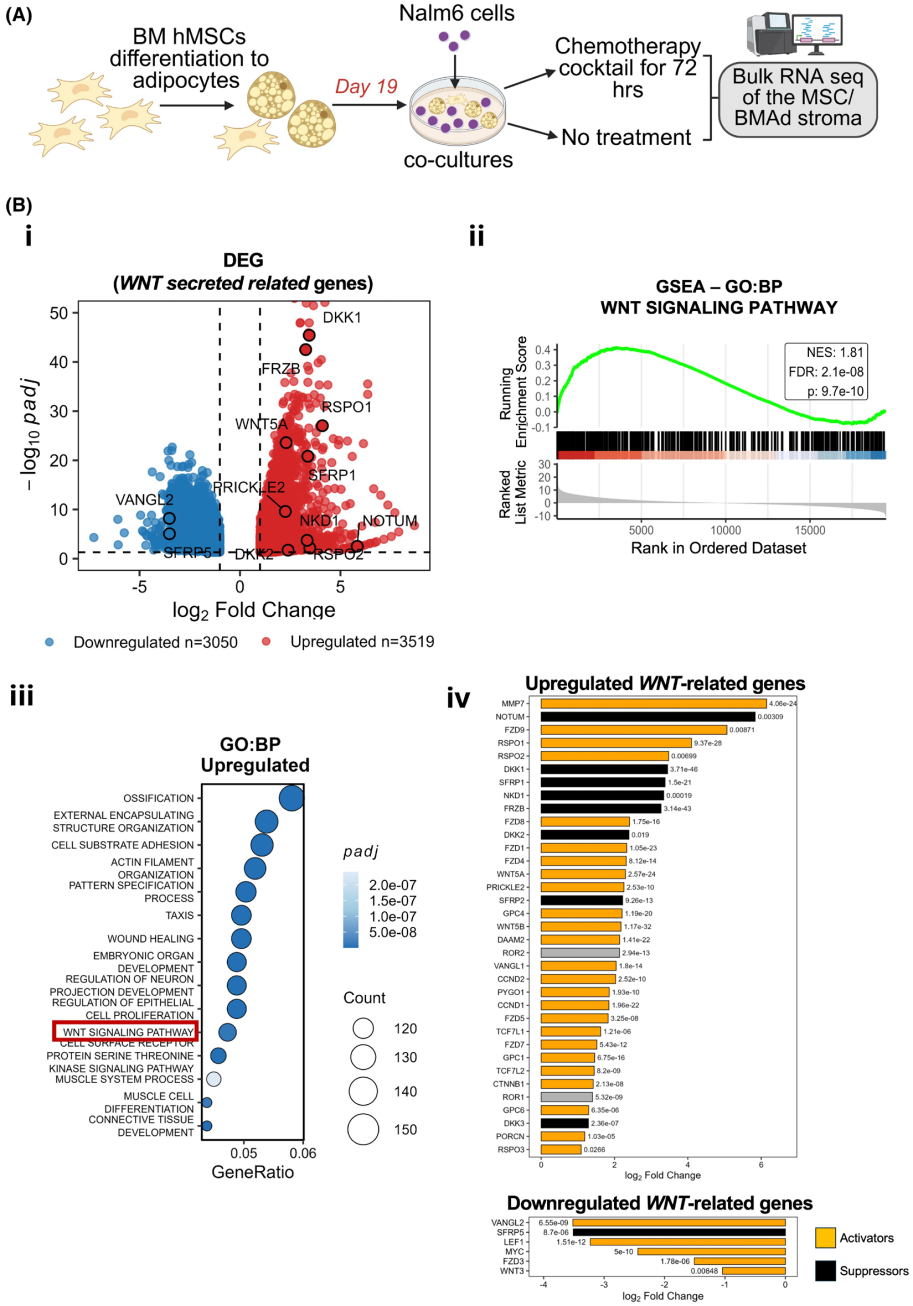
DXV chemotherapy induces WNT Ligands expression in bone marrow‐derived mesenchymal stem cells/adipocytes (MSC/BMAd) stromal cells co‐cultured with Nalm6 ALL cells. (A) Experimental schematic. Nalm6 acute lymphoblastic leukaemia (ALL)cells were co‐cultured with MSC/BMAds and treated for 72 h with DXV (8.3 ng/mL). RNA was isolated from MSC/BMAd stromal cells and transcriptionally profiled. MSC/BMAd stromal cells isolated from untreated co‐cultures served as controls. Samples were derived from four healthy human donors. (B) Transcriptional analysis of MSC/BMAds following DXV treatment. (Bi) Volcano plot showing DEGs (*p*adj < 0.05; log_2_FC >1). Secreted WNT‐related genes are highlighted. (Bii) Gene set enrichment analysis (GSEA) demonstrating enrichment of the WNT pathway in treated stromal cells. (Biii) Over‐representation analysis (ORA) of significantly upregulated gene sets in treated stromal cells, visualised as a dot plot. (Biv) Differentially expressed WNT‐related genes. Bar plots present log_2_ fold changes of upregulated and downregulated WNT‐related genes in treated MSCs/BMAds compared to untreated. Genes are colour‐coded by their function in WNT signalling: Activators (orange), inhibitors (black) and context‐dependent (grey). Adjusted *p*‐values (*p*adj) are indicated.

To reveal niche‐dependent molecular mechanisms driving ALL chemoresistance, we analysed transcriptional changes in MSCs/BMAds isolated after co‐culture with Nalm6 ALL cells under DXV treatment. Mild trypsinisation enabled efficient removal of co‐cultured Nalm6 cells (Figure S2).^15^ mRNAs from untreated and DXV‐treated stromal cells (four donors) were subjected to bulk mRNA sequencing (Figure 2A).

Stromal purity was confirmed based on mRNA expression levels of Nalm6‐associated genes (e.g. *PTPRC, CD10, CD19, CD74, HLA‐DRA, HLA‐DRB1*)^32^, ^33^ and MSC‐associated‐transcripts (e.g. *CD90, CD73, CD105*),^32^, ^34^, ^35^, ^36^, ^37^ which were comparable to those reported in MSC monocultures in previous studies^37^ (Figure S3A). Consistently with adipogenic differentiation, transcripts related to *adipogenesis/BMAds (*e.g. *LEPR, FABP4*)^32^, ^34^, ^35^, ^36^, ^37^ were detected in our MSCs/BMAd stromal cells (Figure S3A). Differential expression analysis revealed significant gene alterations between chemotherapy‐treated and untreated groups (*p*adj < 0.05). Principal component analysis (PCA) showed clear separation of chemotherapy‐stimulated and untreated stromal samples, indicating robust DXV‐induced transcriptional reprogramming (Figure S3B). Heat map analysis of differentially expressed genes (DEGs) further confirmed distinct profiles (Figure S3C). Overall, 3519 genes were upregulated (log_2_FC >1), 3050 downregulated (log_2_FC <−1) and 20 229 were unchanged (Figure 2Bi). Multiple marker genes for MSCs/BMAds^32^, ^34^, ^35^, ^36^, ^37^ were differentially expressed following DXV treatment (Figure S3D). Additionally, gene set enrichment analysis (GSEA) revealed significant downregulation of several gene sets associated with cell division, indicating that DXV markedly affects MSCs/BMAd cells (Figure S3E). This finding is consistent with previous reports describing the inhibitory effect of chemotherapy on MSC expansion capacity.^38^ However, DXV treatment did not affect the viability of treated MSCs (Figure S3F). Importantly, our data showed that major signalling pathways, including PI3K/AKT and WNT, both frequently dysregulated in cancer,^39^, ^40^, ^41^ were enriched following DXV treatment (Figure S2Bii,iii). While WNT signalling is implicated in solid tumours and AML^39^, ^40^ and has been associated with ALL chemoresistance,^11^, ^19^, ^20^, ^21^, ^42^ our results provide, to our knowledge, the first evidence that DXV chemotherapy induces upregulation of WNT ligands at the BM stromal level. Most analysed WNT‐related genes were increased following chemotherapy (Figure 2Biii,iv), including WNT activators such as *FZD1/4/5/7/8/9*, *RSPO1/2/3* and *WNT5B*, as well as repressors (*NOTUM* and *DKK1/3*), many encoding secreted ligands. These findings support a model in which DXV stimulates stromal WNT signalling, promoting paracrine WNT pathway activation in ALL cells, previously linked to ALL chemoresistance.^19^, ^20^

### Pharmacological inhibition of WNT signalling with FH535 abrogates stromal‐mediated chemoprotection and restores apoptotic response in Nalm6 ALL cells

**FIGURE 3:**
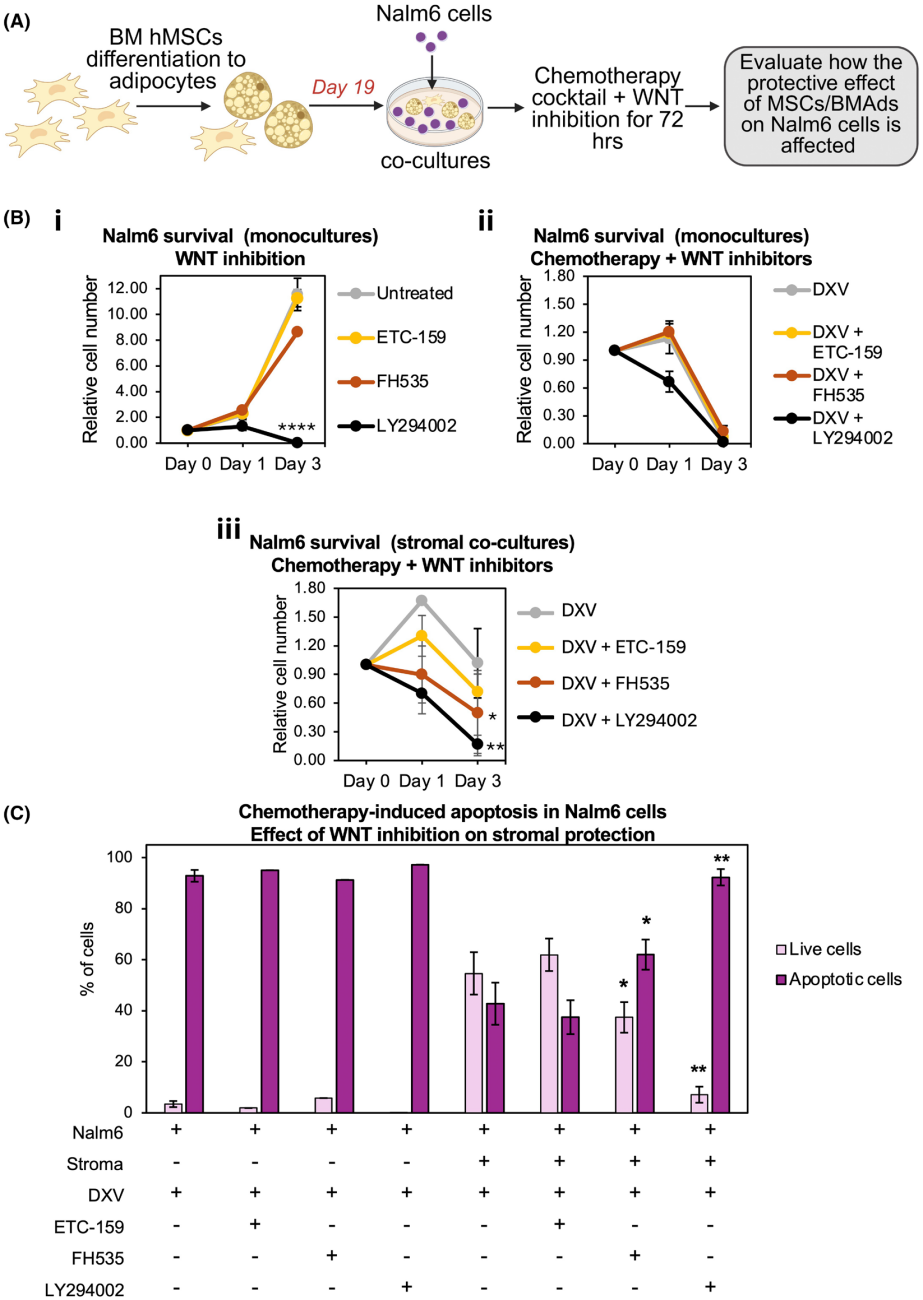
WNT pathway blockade with FH535 sensitises Nalm6 acute lymphoblastic leukaemia (ALL) cells to chemotherapy by disrupting stromal‐mediated survival signals. (A) Experimental schematic. (B) WNT inhibition does not alter Nalm6 cell viability in monoculture but sensitises Nalm6 cells in stromal co‐culture. Quantification of cell survival. Cell numbers were evaluated by manual counting on haematocytometer and normalised to day 0. (Bi) Nalm6 monocultures treated for 72 h with WNT inhibitors (ETC‐159, 100 nM; FH535, 15 μM) or the PI3K inhibitor LY294002 (50 μM). Untreated monocultures served as controls (*n* = 3 technical replicates). (Bii) Nalm6 monocultures treated with DXV alone or in combination with the indicated inhibitors for 72 h. (*n* = 3 independent experiments). (Biii) Co‐cultures of Nalm6 with bone marrow‐derived mesenchymal stem cells/adipocytes (MSC/BMAd) stroma treated with DXV alone or in combination with inhibitors for 72 h (*n* = 3 biological replicates). (C) Apoptosis analysis by flow cytometry using Annexin V and DAPI staining in Nalm6 cells cultured alone or in co‐culture with MSC/BMAds and treated for 72 h as in panel. (B, C) Data represent mean ± SD. ****p < 0.001, **p < 0.01, *p < 0.05.

To assess the functional role of WNT signalling in MSCs/BMAds‐mediated chemoprotection of ALL cells, we pharmacologically inhibited the WNT pathway in vitro using the stromal resistance model and evaluated effects on proliferation and apoptosis in Nalm6 ALL monocultures and ALL–MSC/BMAd co‐cultures (Figure 3).

Two WNT inhibitors were tested: ETC‐159, which blocks WNT ligand secretion via PORCN inhibition,^43^ and FH535, which suppresses β‐CATENIN/TCF‐mediated transcription.^44^ LY294002, a PI3K inhibitor that induces apoptosis in B‐ALL,^41^ served as a positive control (Figure 3A). On‐target WNT pathway inhibition by both inhibitors was confirmed by quantitative reverse transcription PCR (qRT‐PCR) based on downregulation of WNT target genes (*MYC*, *LGR5* and *RNF43*)^11^ (Figure S3G). *AXIN2* mRNA expression (another WNT target) was variable among samples and not significantly downregulated (Figure S3G).

In Nalm6 monocultures, 72‐h treatment with ETC‐159 (100 nM) or FH535 (15 μM) did not significantly reduce cell proliferation compared to untreated controls (Figure 3Bi), whereas LY294002 (50 μM) markedly decreased cell numbers. Similarly, combining WNT inhibitors with DXV did not enhance cytotoxicity, while LY294002 + DXV produced a stronger reduction in cell numbers within 24 h (Figure 3Bii). Thus, WNT inhibition does not potentiate DXV activity in monoculture. In contrast, in MSC/BMAd co‐cultures, co‐administration of DXV with WNT inhibitors accelerated the decline in Nalm6 cell numbers relative to DXV alone (Figure 3Biii), indicating that WNT inhibition disrupts stromal‐mediated chemoprotection. ETC‐159 at 100 nM^43^ was less effective than FH535 (Figure 3Biii), consistent with reports that indicate that higher ETC‐159 concentrations (up to 10 μM) are often required.^23^, ^43^ Thus, likely, responses to ETC‐159 can be improved.

To investigate the underlying cellular mechanism, apoptosis was quantified using Annexin V staining (Figure 3C, Figure S3F). In Nalm6 monocultures, apoptosis remained similar across DXV ± WNT inhibitor treatments. However, in co‐cultures with MSC/BMAd stroma, FH535 + DXV significantly increased apoptosis in Nalm6 cells compared to DXV alone, whereas ETC‐159 + DXV did not, consistent with its weaker effect on cell growth.

Overall, these findings identify WNT pathway activation as a critical driver of stroma‐mediated chemoresistance in ALL and demonstrate that its inhibition can restore chemotherapy sensitivity within the stromal context.

### FH535‐mediated in vivo WNT inhibition enhances the efficacy of TKI therapy in preventing Ph^+^ B‐ALL relapse

**FIGURE 4:**
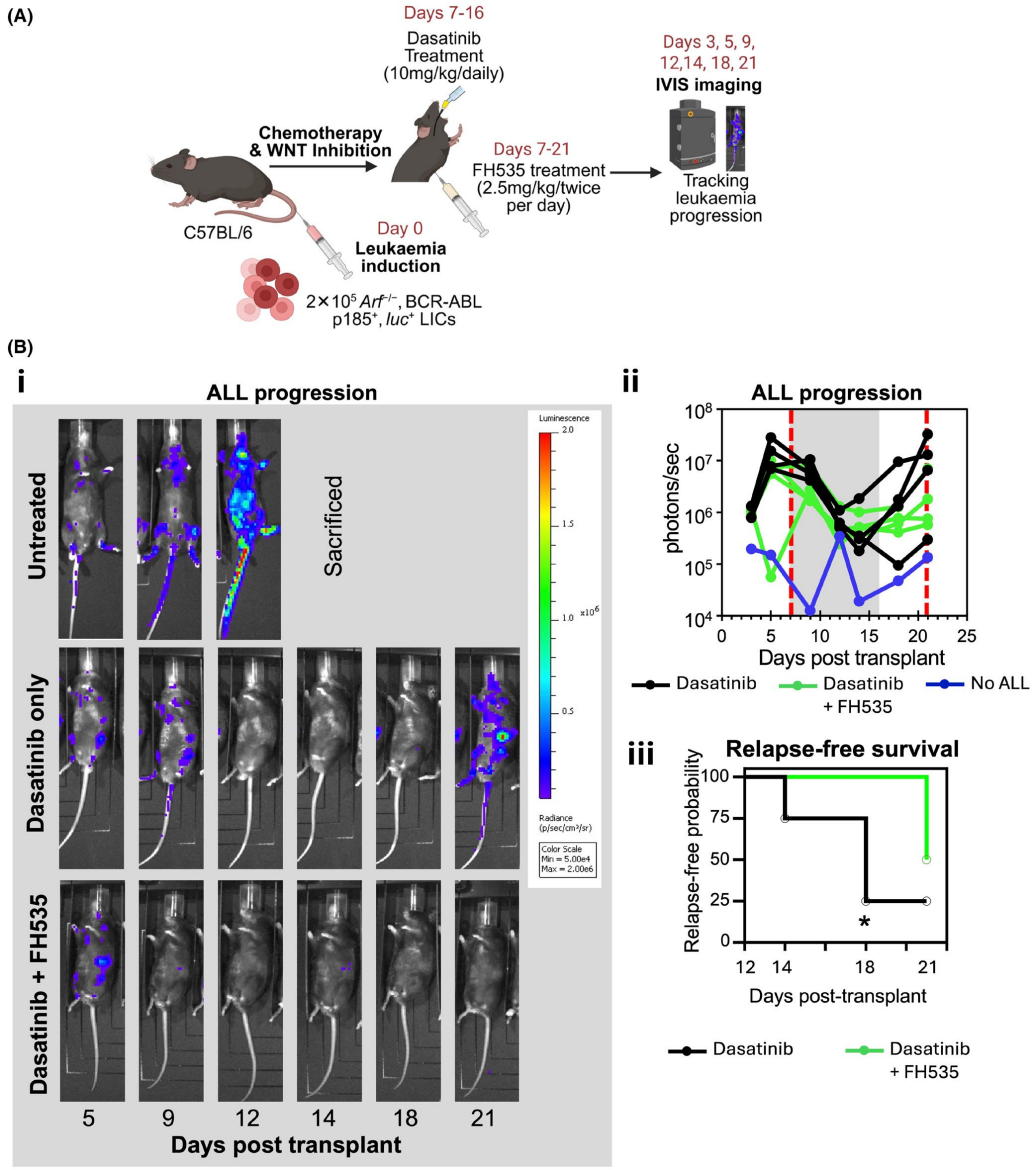
WNT inhibition with FH535 delays relapse in mouse B‐acute lymphoblastic leukaemia (ALL) in combination with TKI. (A) Experimental schematic. C57BL/6 mice were transplanted with *BCR‐ABL* ^+^ *Arf* ^*−/−*^ *Luciferase* ^+^ B‐ALL cells. Leukaemia burden was monitored by in vivo bioluminescent imaging (IVIS) every 3–4 days. On day 7 post‐transplantation, once leukaemia was established, mice were treated with either dasatinib alone (10 mg/kg daily, oral gavage, *n* = 4) or in combination with WNT inhibitor FH535 (2.5 mg/kg, twice daily i.p., *n* = 4). Dasatinib was administered for 10 days, while FH535 was administered until day 21. Mice were sacrificed on day 21. (B) Leukaemia burden and relapse‐free survival. (Bi) Representative IVIS images of individual mice over time. (Bii) Longitudinal whole‐animal bioluminescent signals (photons/s/cm^2^/sr). Grey shading indicates dasatinib treatment period (days 7–16) and red dashed lines confine duration of FH535 treatment (days 7–21). Black lines represent mice treated with dasatinib alone. Green lines depict dasatinib + FH535 treatment. A leukaemia‐free control (blue line) is included for baseline luminescence. (Biii) Kaplan–Meier analysis of relapse‐free survival. Relapse was defined as photon flux >1.3 × 10^6^ after day 12. All dasatinib‐treated mice relapsed during treatment, while mice receiving dasatinib + FH535 remained in remission or relapsed after dasatinib withdrawal. Log‐rank (Mantel–Cox) test revealed statistically significant differences between dasatinib and dasatinib + FH535 curves until day 18 post‐transplant (*p value = 0.0429).

Previous studies showed that WNT inhibition with PRI‐724, a selective β‐catenin/CREB‐binding protein (CBP) interaction antagonist, reduces leukaemic burden in Nalm6 xenografts.^19^ Given our in vitro findings demonstrating that FH535 effectively reversed stromal‐mediated ALL‐chemoprotection in vitro (Figure 3Biii), we next investigated the therapeutic efficacy of FH535 combined with dasatinib in the treatment of ALL in vivo. We used a well‐established B‐ALL mouse model that genetically and phenotypically recapitulates human Philadelphia chromosome‐positive (Ph^+^) ALL,^22^ based on transplantation of Luciferase‐expressing *p185* ^BCR‐ABL^ (p185^+^) *Arf* ^*−/−*^ pre‐B cells^22^ (Figure 4A). Following transplantation of B‐ALL cells into syngeneic immunocompetent mice, this model develops aggressive leukaemia without requiring pre‐conditioning, closely mimicking the progression and pathology of human B‐ALL,^22^ preserving the integrity of the BM niche and avoiding confounding effects associated with irradiation or chemotherapeutic conditioning regimens administered prior to leukaemia development.

Following intravenous transplantation of 2 × 10^5^ B‐ALL cells, all recipients developed leukaemia by day 7^22^ (detectable via bioluminescence in vivo imaging system, IVIS) when treatment was initiated (Figure 4Bi). One cohort (*n* = 4) received dasatinib (10 mg/kg, oral gavage, 10 days^45^), a second‐generation ABL TKI known to induce remission in this model.^22^ A second cohort (*n* = 4) received dasatinib + FH535 (2.5 mg/kg, intraperitoneally, twice daily) until day 21 post‐B‐ALL transplantation (Figure 4B). A non‐transplanted control established background bioluminescence. Relapse, defined after day 12 post‐transplant as exceeding the day 3 maximum baseline signal (1.3 × 10^6^ ph/s), was assessed by IVIS and Kaplan–Meier analysis (Figure 4Bii,iii). While dasatinib monotherapy resulted in relapse during treatment, FH535 co‐treatment either prevented relapse throughout the 21‐day window or delayed onset beyond dasatinib withdrawal (Figure 4Bii,iii). FH535 co‐treatment significantly increased relapse‐free survival up to day 18 post‐transplant (*p*‐value = 0.0429).

These data demonstrate that FH535 enhances dasatinib efficacy by delaying or preventing relapse in vivo, consistent with our in vitro results. This supports WNT pathway inhibition as a BM niche‐targeted strategy to overcome chemoresistance and reduce ALL relapse.

## DISCUSSION

Treatment outcomes for adult ALL remain modest, and relapse, driven by a chemoresistant ALL subpopulation, continues to limit long‐term survival. Identifying therapeutic strategies that eliminate this population is therefore essential. Here, we demonstrated that standard anti‐ALL chemotherapy induces WNT pathway cross‐talk between BM stromal cells and Nalm6 ALL cells by upregulating WNT ligands expression in stromal cells. Pharmacological WNT inhibition reversed stromal‐mediated chemoprotection on B‐ALL cells in vitro. In vivo, WNT inhibition combined with DXV chemotherapy delayed ALL relapse in a murine B‐ALL model. These data support WNT targeting as a strategy to weaken niche‐driven chemoresistance.

WNT signalling is evolutionarily conserved. In the canonical WNT‐β‐CATENIN pathway, WNT ligands bind Frizzled‐LRP5/6 receptors, leading to GSK‐3β/AXIN complex inhibition, β‐CATENIN stabilisation and transcription of targets such as *BIRC5*, *CCND1* and *MYC*.^46^, ^47^ WNT pathway regulates embryogenesis, tissue homeostasis and haematopoiesis, including haematopoietic stem cells (HSC)^11^, ^48^ and lymphocyte biology.^49^, ^50^ Notably, WNT‐β‐CATENIN activation enhances HSC self‐renewal and survival.^48^ Aberrant activation occurs in solid tumours and multiple haematological malignancies including AML, chronic lymphocytic leukaemia (CLL), chronic myeloid leukaemia (CML) and ALL.^51^, ^52^, ^53^ In relapsed childhood ALL, pathway activation arises from promoter hypermethylation of WNT inhibitors and reduced expression of β‐CATENIN/TCF/LEF repressors (e.g. APC, WT1, SOX).^21^, ^54^ Recent work also shows that MSC engagement activates WNT signalling in ALL cells via Integrin‐β1,^19^ promoting an epithelial‐to‐mesenchymal (EMT)‐like transition that contributes to drug resistance.^19^ Consequently, WNT inhibition was shown to increase ALL chemosensitivity.^19^, ^20^

Our findings expand on this stromal–leukaemia interaction by showing that DXV chemotherapy upregulates multiple WNT ligands in BM stromal cells (Figure 2B), implicating the MSC–adipocyte niche as an active driver of WNT activation in ALL cells, which fosters chemoresistance.

MSCs rely on tightly regulated canonical and non‐canonical WNT signalling to sustain their homeostasis, expansion and differentiation.^55^, ^56^ Thus, DXV‐induced perturbation of WNT activity may disrupt fundamental aspects of MSC biology. Such dysregulation could impair their clonogenic capacity and alter lineage commitment, including adipogenic, osteogenic and chondrogenic differentiation,^57^ thereby contributing to niche dysfunction. Moreover, because MSCs regulate HSC self‐renewal, proliferation and differentiation,^11^ disturbances in MSC function are likely to further modify the BM microenvironment. Additionally, chemotherapy is known to damage MSCs in patients with haematological conditions impairing MSC expansion.^38^ Accordingly, our data showed that DXV treatment downregulated gene sets associated with cell proliferation in MSC/BMAds and altered the expression of multiple MSCs/BMAds marker genes (Figure S3D,E).

Functionally, our results indicate that WNT inhibition with FH535 restored chemotherapy‐induced apoptosis in co‐culture (Figure 3Biii,C), confirming that WNT signalling contributes directly to stromal‐mediated protection. Future work should clarify the molecular mechanisms behind DXV‐driven WNT ligand expression; possibilities include activating mutations (e.g. in *WNT1, DKK2, RYK*) and epigenetic silencing of pathway inhibitors.^58^, ^59^ WNT inhibition has previously been shown to resensitise ALL cells to chemotherapy at both tumour‐intrinsic and microenvironment‐dependent levels.^20^, ^21^, ^51^ Several WNT inhibitors, including CBP–β‐CATENIN antagonists, iCRT14, XAV939 and PRI‐724, enhance chemosensitivity in vitro and improve outcomes in xenograft models.^19^, ^20^ Multiple WNT‐targeting therapeutics (e.g. salinomycin, PRI‐724, CWP232291, cirmtuzumab) are in clinical testing for solid and haematological malignancies (including CLL, AML and CML).^60^ Our results reinforce WNT pathway inhibition as a promising approach to counteract ALL chemoresistance in a syngeneic B‐ALL model that closely mimics human disease and preserves the BM niche.

In summary, our work identified chemotherapy‐induced stromal WNT activation as a driver of ALL chemoresistance and demonstrated that WNT inhibition can counteract BM niche‐mediated protection both in vitro and in vivo. These findings provide mechanistic rationale for incorporating WNT inhibitors into current anti‐ALL regimens to target chemoresistant disease, warranting further clinical investigation.

## AUTHOR CONTRIBUTIONS

F.K. designed and performed experiments, analysed data and wrote relevant sections in the manuscript. A.J.‐P. and R.S.‐L. performed and analysed experiments. B.P. conceptualised and designed the study and provided human samples. M.G. conceptualised and designed the study, supervised the project, analysed data, acquired and provided funding and wrote the manuscript. All authors discussed the results and commented on the manuscript.

## FUNDING INFORMATION

M.G. is funded by the American Society of Hematology (Global Research Award, ASH GRA 2021), Kay Kendall Leukaemia Fund (KKL1444), Barts Charity (Research Project Grant G‐002877 & The Rising Stars Programme MGU0459), The Greg Wolf Fund, Leukaemia UK (John Goldman Fellowship, 2020/JGF/001), Blood Cancer UK (25023) and the Medical Research Council (MRC Career Development Award, MR/V009222/1). F.K. is funded by a PhD studentship from Cancer Research UK. B.P. is funded by Blood Cancer UK (15009), The Greg Wolf Fund, Cancer Research UK (A21019), Gabrielle's Angels Foundation.

## CONFLICT OF INTEREST STATEMENT

The authors have no conflicts of interest to disclose.

## ETHICS STATEMENT

All experiments involving mice were performed under Queen Mary University of London Veterinary oversight with UK Home Office authorisation and comply with all relevant ethical regulations regarding animal research. Human samples were acquired from Lonza complying with all ethical regulations.

## MATERIALS AVAILABILITY

No unique reagents were generated in this study.

## DECLARATION OF GENERATIVE AI IN SCIENTIFIC WRITING

During the preparation of this manuscript, the authors used generative artificial intelligence (AI) (ChatGPT) to improve readability and language in some previously written sections of the manuscript. After using this tool, the authors reviewed and re‐edited the content as needed making sure that scientific content and conclusions were accurately preserved and take full responsibility for the content of the publication.

## Supporting information


Data S1.

## Data Availability

Complete RNAseq data are deposited as Sequence Read Archive (SRA) in NCBI servers: PRJNA1381520. All other data in this study are available from the corresponding author on reasonable request.
