# Supplementary material for: Chemotherapy‐driven expression of WNT ligands in bone marrow stromal cells contributes to chemoresistance in acute lymphoblastic leukaemia
Source: Br J Haematol. 2026 Mar 17;208(5):1572–83. doi: 10.1111/bjh.70431 (PMC13176525; doi:10.1111/bjh.70431)
Supplement: Supplementary file 1 — Data S1. [file BJH-208-1572-s001.pdf]

SUPPLEMENTARY MATERIALS

Supplemental Figure 1. Adipogenic differentiation analysis of bone marrow-derived MSCs *in vitro*.

Related to Figure 1A-B. Primary human MSCs derived from healthy donors were differentiated into adipocytes (BMAds) over 19 days. Oil Red O (ORO) staining of lipids in day 19 differentiated MSCs cultures served as a marker of adipogenic differentiation in MSCs. **A.** Representative images are shown in phase contrast from four donors at day 19 of differentiation. Undifferentiated MSCs served as negative controls. Cells were counterstained for 30-60 seconds with Mayer's haematoxylin (Haem). Scale bar: 300 μm. **B.** Quantification of Oil Red O staining as a marker of adipogenic differentiation in MSCs. N = 3 technical replicates per donor from four donors. **B.** Quantification of lipid droplet accumulation in MSC cells. The absorbance of eluted ORO obtained from stained oil droplets was measured in a microplate reader at 492 nm relative to isopropanol elution from unstained cells. Data represent means ± SD. Statistical significance: ****$p < 0.0001$, ***$p < 0.001$, **$p < 0.01$, *$p < 0.05$.

Supplemental Figure 2. Nalm6 and MSC/BMAds isolation from Nalm6-MSC/BMAds co-cultures.

Related to Figure 1D and 2. **A.** Flow cytometry analysis of Nalm6 and MSC/BMAds cell populations recovered by mild trypsinisation from Nalm6 + MSC/BMAds co-cultures. **A.** Representative flow cytometry plots depicting Nalm6 and MSC/BMAds mono-cultures and Nalm6 + MSC/BMAds co-cultures. Additionally, Nalm6 and MSC/BMAds cells

isolated by mild trypsinisation post co-culture are shown. CD45 was used as a pan-hematopoietic marker to confirm the identity of Nalm6 cells. **B.** Ratio of Nalm6 cell numbers over MSC/BMAds numbers was used as a measure of population purity in MSC/BMAd (MSC/Ads) and Nalm6 recovered post-co-culture (untreated or treated with DXV at IC50 or IC90). MSC/BMAds monocultures and Nalm6 + MSC/BMAd co-cultures are depicted as controls. Kruskal Wallis test with Dunn's multiple comparisons were applied as data was not normally distributed. Differences with MSC/BMAds mono-cultures are shown. Data represent means ± SD. Statistical significance: ****$p < 0.0001$, ***$p < 0.001$, **$p < 0.01$, *$p < 0.05$.

Supplemental Figure 3. DXV chemotherapy induces transcriptional reprogramming in BM MSCs and BMAds. A-F. Related to Figure 2. Transcriptional characterization of MSC/BMAds isolated from four healthy human donors co-cultured with Nalm6 ALL cells and exposed to DXV (8.3 ng/mL) for 72 hours. **A.** Stromal purity of recovered MSCs/BMAds was assessed. Normalized read counts for housekeeping-, adipocytic-, BMAds-, MSC- and Nalm6-related transcripts are shown (blue columns). As reference, normalized read counts from the GSE212209 dataset[37], comprising MSC monocultures and MSCs recovered after 72-h co-culture with Nalm6 cells, are shown. Transcript levels were overall comparable to MSC monocultures for all genes, except for adipocytic markers, which appear higher in our samples because MSCs in GSE212209 were not differentiated, whereas adipogenesis was induced prior to co-culture in our study. Comparisons among studies are semi-quantitative as data from both studies were not normalized together and

direct statistical comparisons are not possible. **B.** PCA was performed on variance-stabilised counts. Red dots indicate DXV-treated samples; blue dots represent untreated controls. Each donor is shown with a unique shape. PC1 (87% variance) distinguishes treated from untreated samples. PC2 (6% variance) captures inter-donor variation. **C.** Heatmap of the 500 most variable genes across all samples. Columns represent individual stromal samples grouped by treatment; rows display genes. Relative expression levels are colour-coded (red = high, blue = low). **D.** DXV treatment perturbed the expression of MSC/BMAds marker genes. Normalized read counts are shown. **E.** GSEA demonstrated downregulation of cell division-related gene sets in MSC/BMAds **(Ei)**. Top-15 downregulated cell cycle/replication genes are shown **(Eii)**. **F.** Related to Figure 3C. **Fi.** Flow cytometry gating strategy used to identify apoptotic cells based on Annexin V and DAPI staining. **Fii.** Apoptosis analysis by flow cytometry using Annexin V and DAPI staining in undifferentiated MSC cells cultured alone and DXV-treated (8.3 ng/mL) for 72 hours (n=3-4 biological replicates from different donors). **G.** Analysis of on-target WNT pathway inhibition. mRNA expression levels of WNT target genes (*MYC, LGR5, AXIN2 and RNF43)* was evaluated via qRT-PCR on MSC/BMAds isolated from four healthy human donors co-cultured with Nalm6 cells and exposed to DXV (8.3 ng/mL) for 72 hours. Standard deviations show variation among biological replicates (n=3). Transcript levels were normalized to those in untreated cells for each donor (=100). Means and standard deviations are indicated. **** $p < 0.001$, *** $p < 0.001$, ** $p < 0.01$, *$p < 0.05$.

Supplemental Table 1: Key Reagents

Reagent or resource	Source	Identifier
**Antibodies**		

Annexin V-APC	Biolegend, San Diego, CA	Cat # 640941
Anti-mouse/human Ki67-APC	Biolegend, San Diego, CA	Cat # 151231
Donkey anti-goat IgG-FITC	SouthernBiotech	Cat # 6420-02
Goat anti-human FABP4	R&D Systems	Cat # AF3150
Anti-human CD45-BV421	BioLegend	Cat # 304032
**Experimental models: organisms/strains/cell lines**		
C57BL/6NCrl	Charles River	Stock #027
Nalm6	ATCC	CRL-3273
Arf$^{-/-}$, BCR::ABL-p185^{+}, *luc*$^{+}$ pre-B cells	St Jude Children's Research Hospital (Memphis, TN, USA)	
Primary human-BM-MNCs	Lonza	2M-125C
**Software and algorithms**		
GraphPad Prism 9	GraphPad Software, Boston, Massachusetts	
FlowJo version 10.10	BD Life Science	
**Chemicals, peptides, and recombinant proteins**		
FH535	Sigma-Aldrich	#219330 (Lot: 3942733)
ETC-159	Cayman Chemical Company	#24104 (Lot: 0540730-11)
LY294002	Cell Guidance Systems	SM24-10 (Lot: 0517)
MEMα nucleosides	Gibco™	12571063
FBS MSC-qualified	Gibco™	11548986
Adipogenesis kit	Lonza	PT-3004
Dasatinib	Cell guidance systems	SM45
VivoGlo™ Luciferin, *in vivo* grade	Promega	P104C (Lot: 0000564312)
DAPI	BD Biosciences	#564907
Annexin V Binding Buffer	STEMCELL technologies	#100-0334
**Oligonucleotides**		
Name	Sequence (5'- 3')	
AXIN2-Human-Fw	CAAACTTTCGCCAACCGTGGTTG	
AXIN2-Human-Rv	GGTGCAAAGACATAGCCAGAACC	
CCND1-Human-Fw	TCTACACCGACAACTCCATCCG	
CCND1-Human-Rv	TCTGGCATTTTGGAGAGGAAGTG	

c-MYC-Human-Fw	CCTGGTGCTCCATGAGGAGAC
c-MYC-Human-Rv	CAGACTCTGACCTTTTGCCAGG
LGR5-human-Fw	CCTGCTTGACTTTGAGGAAGACC
LGR5-human-Rv	CCAGCCATCAAGCAGGTGTTCA
RNF43-Human-Fw	GGTTACATCAGCATCGGACTTGC
RNF43-Human-Rv	ATGCTGGCGAATGAGGTGGAGT
GAPDH-Fw-1	GTCTCCTCTGACTTCAACAGCG
GAPDH-Rv-1	ACCACCCTGTTGCTGTAGCCAA

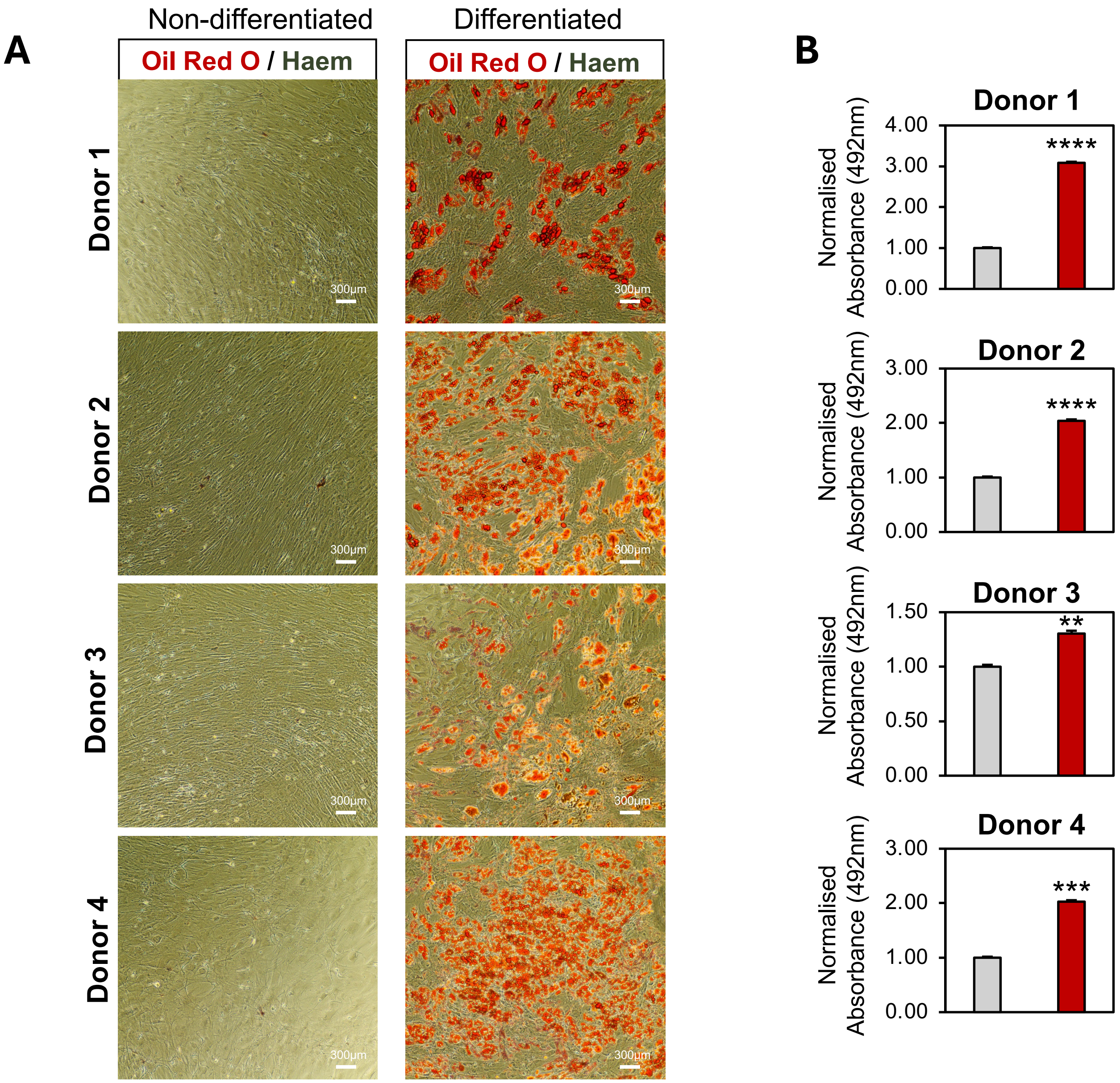

Supplementary Figure 1

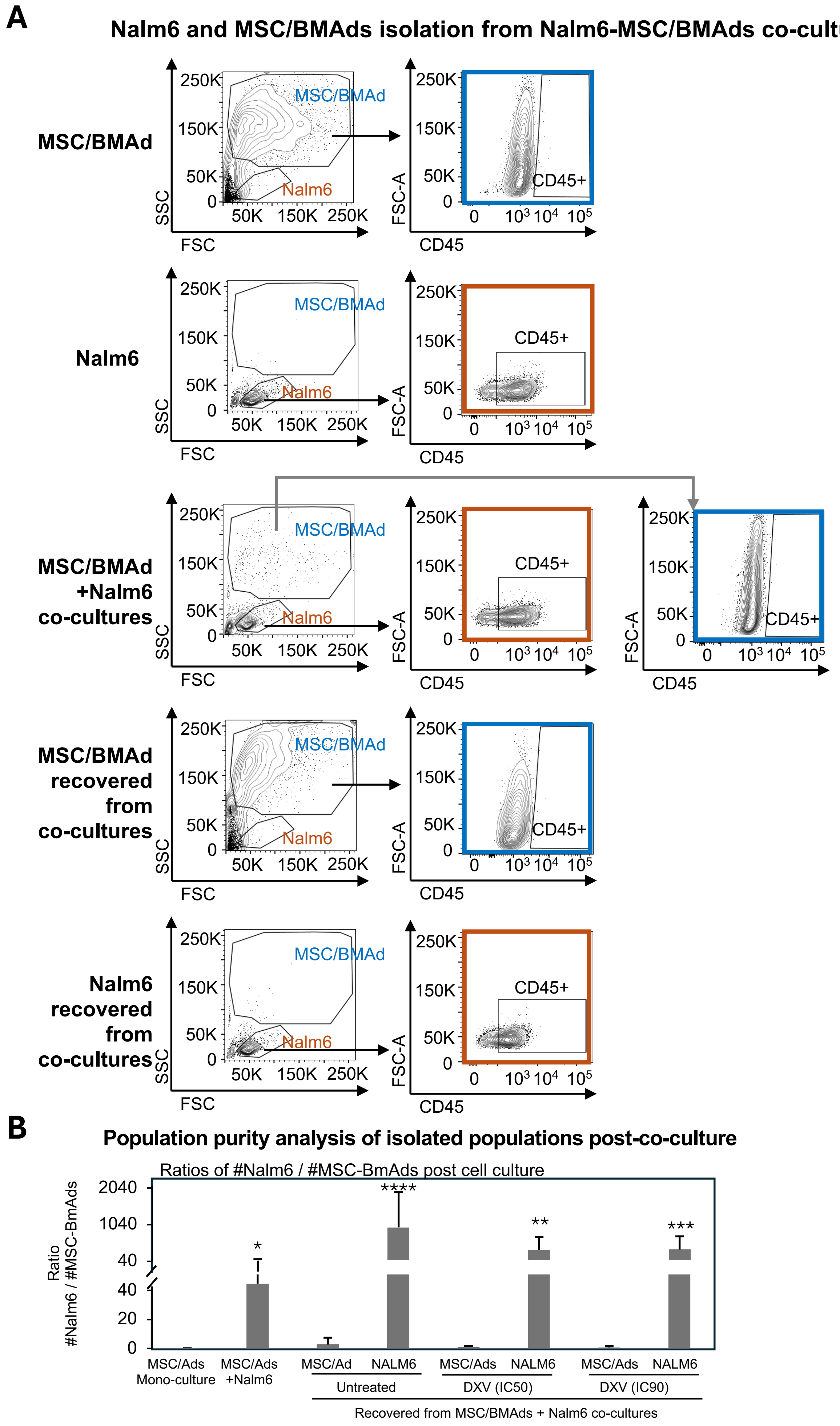

Supplementary Figure 2

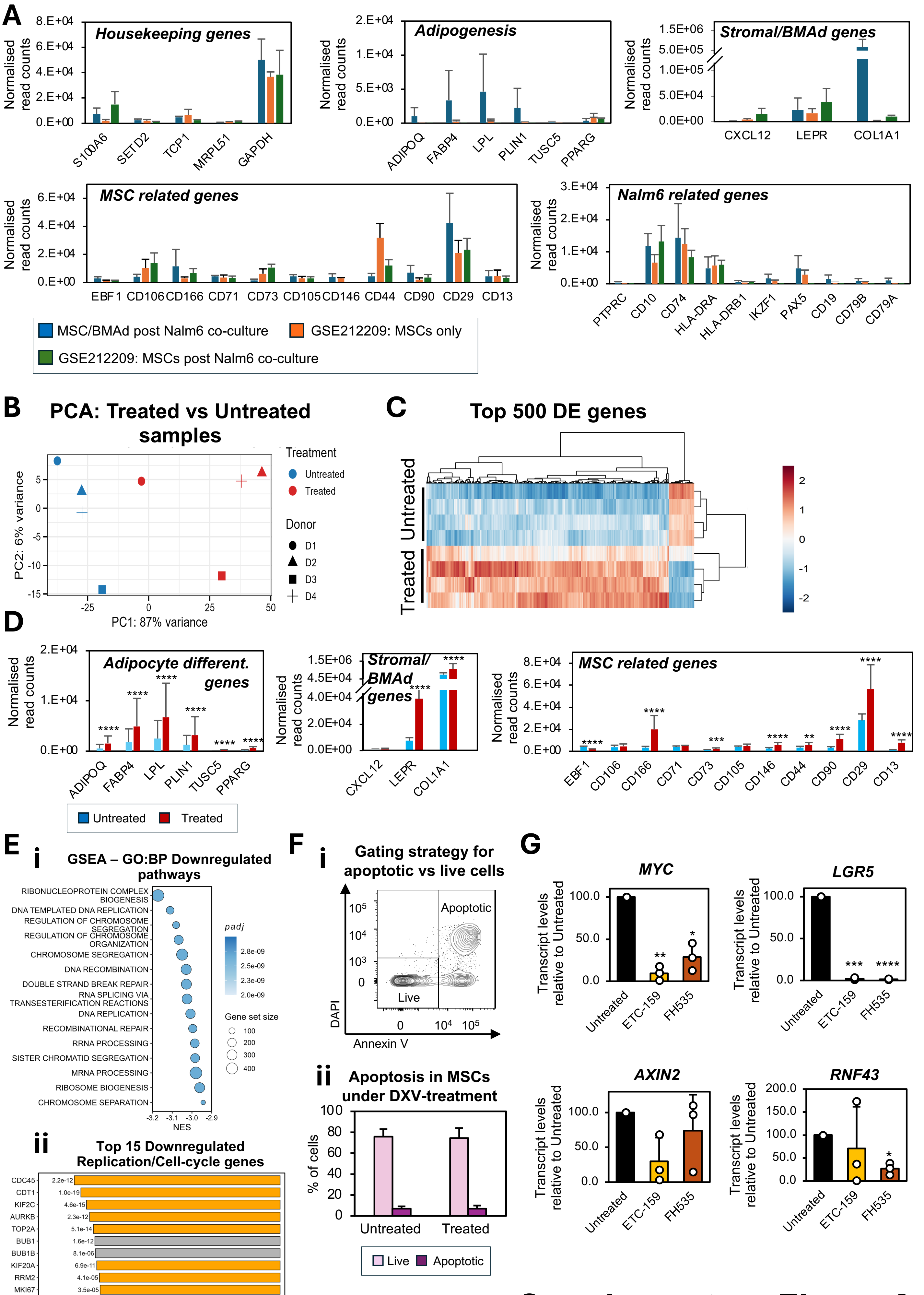

Supplementary Figure 3
